# Diet, sex, and death in field crickets

**DOI:** 10.1002/ece3.288

**Published:** 2012-07

**Authors:** Felix Zajitschek, Simon P Lailvaux, Josephine Dessmann, Robert Brooks

**Affiliations:** 1 Department of Animal Ecology, Evolutionary Biology Centre, Uppsala University75236 Uppsala, Sweden; 2 Department of Biological Sciences, The University of New Orleans2000 Lakeshore Drive, New Orleans, Louisiana 70148; 3 Evolution & Ecology Research Centre and School of Biological, Earth and Environmental Sciences, The University of New South WalesKensington, Sydney 2052 NSW, Australia

**Keywords:** Ageing, calling effort, Gompertz, senescence, sex differences, *Teleogryllus commodus*

## Abstract

Senescence is shaped by age-dependent trade-offs between fitness components. Because males and females invest different resources in reproduction, the trade-offs behind age-dependent reproductive effort should be resolved differently in the sexes. In this study, we assess the effects of diet (high carbohydrate and low protein vs. equal carbohydrate and protein) and mating (once mated vs. virgin) on lifespan and age-dependent mortality in male and female field crickets (*Teleogryllus commodus*), and on male calling effort. Females always had higher actuarial ageing rates than males, and we found a clear lifespan cost of mating in females. Mated males, however, lived longer than virgin males, possibly because virgins call more than mated males. The fastest age-dependent increases in mortality were among mated males on the high-carbohydrate diet. Males on a high-carbohydrate diet showed a faster increase in calling effort earlier in life, and a more pronounced pattern of senescence once they reached this peak than did males on a diet with equal amounts of protein and carbohydrates. Our results provide evidence that the cost of mating in this cricket species is both diet and sex-dependent, and that the underlying causes of sex differences in life-history traits such as lifespan and senescence can be complex.

## Introduction

Finite resources allocated to one fitness-related trait cannot be used for another, enforcing trade-offs in the timing and quantities of resources allocated to various fitness-related traits (Stearns 1992). The timing of allocation is particularly important because stochastic extrinsic mortality causes the strength of selection operating on a cohort to weaken as that cohort ages, reducing the likely fitness return of the same quantum of investment in reproduction as an individual ages (Medawar 1952; Williams 1957). Males and females may invest different resources in reproduction, and the costs of male and female reproductive effort can also differ. As a result, the optimum timing and magnitude of resource allocation to reproduction might be expected to vary dramatically between males and females. In this paper, we explore sex differences in lifespan and ageing by experimentally manipulating both reproduction (by altering mating status) and resource acquisition (by altering diet quality).

The timing of life-history trade-offs can be greatly affected by the pool of resources an individual has acquired (i.e., the individual's condition (Rowe and Houle 1996; Hunt et al. 2004b). While variation in condition is at least partially genetic (Blanckenhorn and Hosken 2003), it is possible to experimentally manipulate condition by manipulating diet components (Hill 2011). This has important implications for ageing because if diet (and hence condition) varies among individuals then we might expect those individuals in good condition to be able to sustain somatic maintenance and reproductive effort for longer than individuals in worse condition can, thus postponing the onset of senescence. Alternatively, good-condition individuals might invest more heavily early in life compared with individuals in poorer condition, elevating mortality risks or somatic damage such that senescence is accelerated. Males and females are known to differ in the resources they use for reproduction (Masoro and Austad 2006) and in the ways in which they can discount the future in relation to the anticipated costs of reproduction. This live-fast die-young strategy (Vinogradov 1998) is hypothesized to be more common in males than in females in many species where sexual selection on males is strong and males discount the future to invest heavily in sexual traits or territorial dominance (e.g., wolf spiders, Kotiaho et al. 1996; humans, Wilson and Daly 1997; crickets, Hunt et al. 2004a).

Mating and reproduction impose costs on both sexes, and many of these costs are likely to be different for males and females. Costs of mating and reproduction may also be lessened or amplified by individual condition. In males, theory predicts that sexual signals of genetic quality should be condition-dependent, with high-quality males best able to bear the costs of signaling (Zahavi 1975; Pomiankowski 1987; Grafen 1990). Indeed, females in many taxonomic groups often choose mates on the basis of energetically costly sexual signals (e.g., acoustic signals; Ryan 1988). Finding, courting, and copulating with a mate might all be energetically costly and raise the risk of predation (Zuk and Kolluru 1998), but the costs of mating do not end there. For example, in water striders, the actual energetic cost for females of mating (and even more so of premating struggles against male harassment) is higher than even those for locomotion (Watson et al. 1998). In *Drosophila melanogaster*, male ejaculate includes proteins that negatively affect female survival and displace the females’ egg laying and re-mating rates from their optima (Chapman et al. 1995). These effects in female *D. melanogaster* have been shown to be diet-dependent (Chapman and Partridge 1996; Fricke et al. 2010). More generally, the availability of sufficient energetically rich macronutrients in the diet should ameliorate many physiological costs of mating.

**Figure 1:**
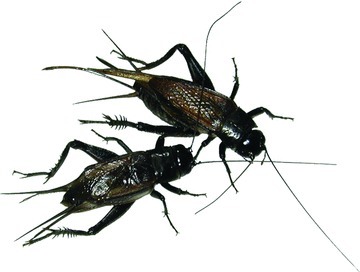
A male (bottom) and a female (top) black field cricket, *Teleogryllus commodus*.

Given the striking differences in the nature of reproductive effort between males and females of most species, it is likely that the sexes differ in the nutritional target, that is, the amount of nutrients that would best optimize fitness. In *Teleogryllus commodus* (Fig. 1), the dietary macronutrient requirements that optimize fitness are sex-specific: males do best on more energy-rich diets as calling is energetically demanding, whereas females require more protein for egg production (Maklakov et al. 2008a). In our previous studies (Maklakov et al. 2008b, 2009), however, only mated crickets were used, leaving us unable to establish the effects of mating on the sex- and diet-dependent patterns of ageing that we found. In the present study, we tested for the effects of mating, diet, and their interaction on mortality patterns in *T. commodus* males and females, and on calling effort in males. We predicted that mated females will die earlier than virgin females, if there is a direct cost of mating and egg production. We also predicted females on the diet containing more protein would cope better with the resource costs of reproduction, resulting in lower mortality rates. Our prediction for mated males was that they would die sooner and senesce more rapidly than virgin males due to the costs of mating, and we measured not only actuarial senescence but also senescence in male calling in order to test this prediction. Our prediction regarding the costs of mating was tempered, however, by the possibility that virgin individuals might call more than mated males, thus altering our prediction regarding senescence. Calling might even lead to a shorter lifespan and higher mortality or ageing rates of virgin males, with the magnitude of the difference between virgins and mated males depending on the actual cost of mating compared with the cost of the difference in calling. We predicted that males on a diet high in carbohydrates would call more compared to males on a lower carbohydrate diet. Our study also allows us to detect more complex interactions between diet, mating status, reproductive effort, and ageing. Finally, based on results from previous studies (Zajitschek et al. 2009a,c), we predicted that females would show a higher ageing rate in terms of mortality than males.

## Methods

### Experimental design and diets

We used a 2 × 2 × 2 design, in which we manipulated diet (two levels) and mating status (two levels) in both males and females, resulting in four treatments: diet C/virgin (CV), diet C/mated (CM), diet P/virgin (PV), diet P/mated (PM). We manipulated resource acquisition by providing experimental crickets with one of two artificial diets. Diet C was rich in carbohydrates, with a protein-to-carbohydrate ratio (P:C) of 1:8. Diet P contained equal amounts of protein and carbohydrates. (The exact percentages of dietary components were 9.33:74.66 P:C for diet C, and 42:42 for diet P, both adding up to the same protein and carbohydrate content of 84% in each diet. The remaining 16% consisted of indigestible crystalline cellulose.) We made artificial diets according to an established protocol (Simpson and Abisgold 1985). Proteins consisted of a 3:1:1 mixture of casein, albumen, and peptone, and digestible carbohydrates were a 1:1 mixture of sucrose and dextrin. All artificial diets contained Wesson's salts (2.5%), ascorbic acid (0.275%), cholesterol (0.55%) and a vitamin mix (0.18%).

We experimentally assigned each individual to one of two mating status treatments: either virgin (V; unmated) or mated (M; a cricket was mated overnight with a different random stock culture individual of the opposite sex every seven days until it died). On the day of their eclosion into adulthood, we isolated experimental crickets of both sexes from our laboratory stock cultures from Smith's Lake (NSW, Australia) that were fed a diet of cat food (Friskies GoCat Senior) and water. Freshly eclosed crickets were kept from then on until they died in individual plastic containers (5 × 5 × 5 cm) that contained a piece of egg carton for shelter and a plastic water bottle plugged with cotton wool. We assigned individuals alternately to one of the four treatments (sample sizes males: CV = 82, CM = 76, PV = 72, PM = 70; females: CV = 71, CM = 76, PV = 61, PM = 67). On the day of eclosion (adult age = 0 days), pronotum width was measured using an eyepiece graticule in a binocular microscope (Leica MZ5), and animals were weighed on an electronic balance (Mettler-Toledo, model AG135) to the nearest 0.0001 g. Plastic containers were cleaned and new diet and water was provided weekly. Survival was checked daily.

### Reproductive effort

Male reproductive effort was measured as each male's nightly calling effort—defined as the amount of time a male broadcasts its long distant sexual advertisement call (between 1800 and 0900 h). We measured each male cricket from four days post eclosion, every five days, until its death. Data recording was accomplished with a custom built electronic event recorder that sampled 10 times per second whether or not a given male cricket called. Microphones were mounted in each lid of the individual cricket containers (for technical details, see supplement of Hunt et al. 2004a).

Mated females were given the opportunity to lay eggs in moist sand, provided in petri dishes, but numbers of eggs were not counted in the present study due to logistic constraints. If suitable substrate is provided, unmated females in this species lay eggs, but in quantities much smaller than those laid by mated females (Loher and Edson 1973). In the present experimental design, unmated females were not provided with egg-laying substrate and therefore laid a negligible number of eggs (ranging from 0 to 10) during their lifetimes. This means that in females, we were able to study the survival cost of reproduction, by comparing virgin and mated females, but we could not estimate age-dependent and lifetime female fecundity.

### Statistical methods

We modeled the effects of diet, sex, and mating treatment on male calling effort using linear mixed-effects models, using the function lme of package nlme (Pinheiro et al. 2009), available in the software R (R Development Core Team 2009). The full model included three-way interactions diet × mated × age, diet × mated × age^2^, two-way interactions lifespan × diet, lifespan × mated, lifespan × age, lifespan^2^× diet, lifespan^2^× mated, lifespan^2^× age and all lower order terms. Cricket identity was fitted as a random effect to account for temporal autocorrelation (van de Pol and Verhulst 2006). Model selection was achieved by comparing Akaike's information criterion (AIC), with the best-fit model having the lowest AIC value. The difference between a model's AIC and the best-fit model's AIC is referred to as ΔAIC. Models with ΔAIC smaller than 2 are assumed to provide equivalent fit and have substantially more support than models with AIC >2 (Burnham and Anderson 2002). The best-fit model for male calling effort was substantially better than all other candidate models (ΔAIC of the next best model = 3.4); therefore, we present model results of the best-fit model only. To visualize treatment effects on calling effort, we created nonparametric thin-plate spline contour plots with the function Tps from the package fields in R (Furrer et al. 2012).

To model age-dependent mortality rates and test for treatment effects, we fitted five commonly used mortality models (Gompertz, Gompertz-Makeham, Logistic, Logistic-Makeham, and Weibull) to the data. For this, we used maximum likelihood fitting methods implemented in the R package bbmle (Bolker 2012). Models with a Weibull distribution are not nested within candidate models that include the other four distributions. Therefore, we used AIC for model selection.

None of the other four tested distributions provided a substantial improvement in fit on the Gompertz models. Because the Gompertz model is the least complex, it is therefore the most parsimonious of all tested models, and we proceeded to test the direct and interaction effects of sex, mating treatment, and diet on model parameters within the Gompertz models only. The Gompertz mortality model is defined as


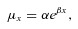


where μ*_x_* is the age-dependent instantaneous rate of mortality, also referred to as hazard rate or force of mortality. This measure of age-dependent mortality in any specific age interval is not influenced by individuals dying in the intervals preceding or following this interval. The parameter α is interpreted as the baseline mortality rate, and β as the rate of increase in mortality rate in late ages, also called the “rate of ageing” parameter of actuarial ageing. We estimated the importance of treatment effects (diet, mated, diet × mated) on a Gompertz parameter, that is, how well the data supported a specific treatment effect, by fitting 21 models that covered all possible combinations of effect terms on Gompertz α and β (see Tables S1 and S2 for full model specifications). In addition, we also included a “No senescence” model that modeled a constant age-independent hazard rate (μ_1_*x* = α). For the maximum likelihood estimations, models were linearized by log-transformation. Model fits were compared using AIC values. To further interpret ΔAIC, we calculated Akaike weights which provide a measure of the weight of evidence that a specific model, of all tested models, is the best-fit model (Burnham and Anderson 2002). We compared models separately for each sex, as a likelihood ratio test (with twice the difference in log-likelihoods assumed to follow a χ^2^ distribution) between the full model (containing the effects of diet × mated × sex and lower order terms on both Gompertz parameters) and the full model without sex showed a better fit of the former (χ^2^ = 17.94, df = 8, *P* = 0.022).

## Results

### Mortality

**Figure 2:**
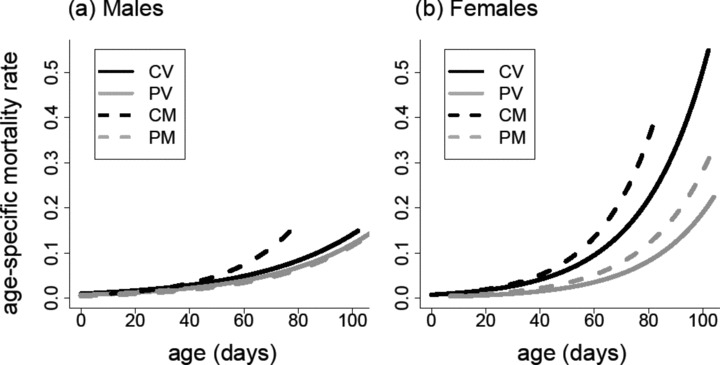
Gompertz mortality trajectories of treatment groups of males (a) and females (b). Please note that these curves correspond to models fit separately to the four treatment groups (see Fig. S1 for parameter estimates), and do not represent any selected best-fit models. Lines end at realized maximum lifespans.

Females and males in all treatment groups exhibited a senescent increase in mortality rate late in life (“No senescence” models in Tables S1 and S2). Females had consistently higher ageing rates (β) than males, independent of treatment (Table 1). In females, diet and mating treatment substantially affected both α and β (models 1–4 in Table S1). Females on diet P had lower baseline mortality than females that were fed diet C (Fig. 2; Table 1) and virgin females aged slower than mated females (Fig. 2; Table 1). There was no evidence for interaction effects between treatment variables on α and on β (in Table S1, model 2 with the mated × diet interaction term provided an equal fit compared to model 1, without this interaction).

**Table 1:** Gompertz parameter estimates for male and female mortality models. Values in brackets are lower and upper 95% confidence intervals

	Males		Females	
		
Treatment	α	β	α	β
CV	0.0101 (0.0064, 0.0153)	0.0264 (0.0171, 0.0356)	0.0078 (0.0045, 0.0128)	0.0417 (0.0291, 0.0543)
CM	0.0065 (0.0038, 0.0107)	0.0406 (0.0290, 0.0522)	0.0075 (0.0046, 0.0118)	0.0482 (0.0364, 0.0597)
PV	0.0073 (0.0044, 0.0116)	0.0280 (0.0189, 0.0370)	0.0029 (0.0028, 0.0030)	0.0418 (0.0414, 0.0423)
PM	0.0059 (0.0035, 0.0096)	0.0299 (0.0209, 0.0387)	0.0041 (0.0023, 0.0071)	0.0423 (0.0318, 0.0526)

In males, diet had the most substantial effect on α and on β (models 1–3 in Table S2) and there was evidence that mating treatment had explanatory power (models 4–7 in Table S2). Mated males fed diet C had the highest ageing rate, whereas males in the other three treatment groups showed similar ageing rates (Fig. 2; Table 1). This result is also substantiated by the importance of the interaction term mated × diet on β (models 5 and 7 in Table S2). Virgin males had a slightly higher baseline mortality compared to mated males, independent of diet (Table 1).

With median lifespan as an indicator of longevity, mated males lived longer than virgin males, in contrast to females where we found the opposite (Table 2). Maximum lifespan was defined as median lifespan of the longest lived 10% in each group. It was shortest for mated males on diet C, owing to the high ageing rate in this group. In both sexes, animals on diet C lived on average shorter than those on diet P.

**Table 2:** Median and maximum lifespan of treatment groups in days. Values in brackets are bootstrapped lower and upper 95% confidence intervals (based on 5000 bootstrap replicates). Maximum lifespan was defined as median lifespan of the longest lived 10% in each group (median [upper 10%])

	Males		Females	
		
Treatment	Median	Median (upper 10%)	Median	Median (upper 10%)
CV	36 (27, 39)	84	37 (32, 46)	76.5
CM	42 (36, 48)	75	35 (31.5, 38)	62
PV	43.5 (31, 49)	88	55 (45, 61)	87.5
PM	49 (37, 56)	90	51 (45.5, 58)	78.5

### Calling effort

**Figure 3:**
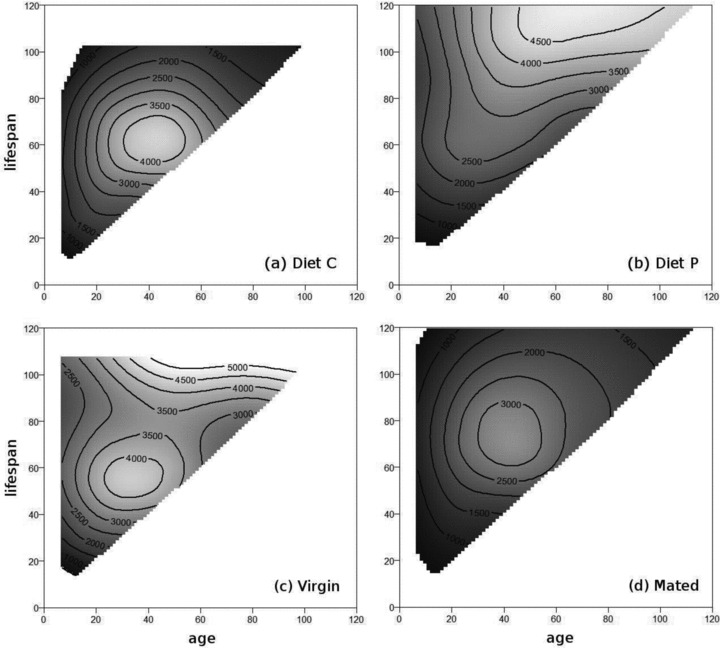
Calling effort (seconds per night) of males on diet C (a) and males on diet P (b), and of virgin (c) and mated (d) males. Lifespan and age are given in days of adult life.

The estimated best-fit model parameters for male calling effort are given in Table 3. The diet × mating treatment interaction and all diet × mating treatment × covariate interactions do not form part of this model, indicating that the effects of diet and mating on calling effort and on age-dependent and lifespan-dependent ageing are independent. In fact, the only treatment × covariate interactions in the best-fit model are diet × age and diet × age^2^, suggesting that diet alters age-dependent calling trajectories (Fig. 3A and B), but mating status does not (Fig. 3C and D). The diet-induced difference in age-dependent calling comes about because males on the carbohydrate-rich diet (C) reach a peak calling effort at about 40 days of age and then senesce, whereas those on the equal protein and carbohydrate diet steadily increase their calling effort, with only the longest lived males reaching a plateau. Because of the interaction term between diet and age in the best-fit model, we cannot directly infer whether overall calling effort of males on different diets differed on average. Additional tests in both mating groups showed no such difference (virgin males: *t*(100.85) = −0.85, *P* = 0.40; mated males: *t*(119.84) = 0.66, *P* = 0.51); that is, average calling effort was not higher in males on diet C compared to diet P, contrary to our prediction.

**Table 3:** Best-fit model results for male calling effort. Baseline category for diet is diet C and mated for mating treatment; that is, the reported values give the estimated change in calling effort between baseline category and the category named in the table

Model term	Coefficient	SE
Diet (P)	1299.3	752.1
Mating treatment (virgin)	1059.1	451.1
Age	95.4	35.7
Lifespan	78.3	44.1
Age^2^	− 3.2	0.5
Lifespan^2^	−1	0.4
Diet (P): age	−123.6	40.6
Diet (P): age^2^	1.4	0.5
Age: lifespan	2.4	0.5

On average, virgin males called more than mated males, independent of the diet they received and independent of male age (see coefficient for mating treatment in Table 3; Fig. 2C and D).

## Discussion

Both diet and reproductive effort can have important effects on resource allocation toward somatic maintenance and subsequent reproduction. These effects on allocation alter both actuarial and reproductive ageing. Because the types of investments that males and females make differ, we predicted that ageing patterns should often also differ between the sexes. We found that males aged much more drastically in late life on a high-carbohydrate diet, compared to males on the equal P:C diet, but only if they were mated. The finding that virgin males called more coincides with a slightly higher baseline mortality rate in these males. Female survival at first sight appears to exemplify a classic cost of reproduction, but further parametric mortality modeling revealed an interaction between diet and mating status. Specifically, mated females on the high-carbohydrate diet aged more rapidly late in life (higher Gompertz β) than virgin females, whereas on equal P:C diet, age-independent baseline mortality (Gompertz α) of mated females was higher, compared to virgin females.

Generally, female field crickets showed more rapid actuarial ageing than males. This effect was not dependent on their diet or on their mating status. Consistent with previous studies (Hunt et al. 2004a; Maklakov et al. 2008a), life expectancy was lower on the carbohydrate-rich diet, especially for females, and females also paid a cost of mating (and subsequent reproduction) in terms of lower life expectancy and maximum lifespan, independent of diet. Strikingly, the mortality trajectories that led to these lifespan patterns depended on diet. Mated females on the carbohydrate-rich diet aged more rapidly late in life than virgin females, while maintaining the same baseline mortality compared to virgins. On the other hand, mated females on the equal P:C diet had a similar late-life ageing rate to virgin females, but a higher baseline mortality. These patterns might point to an effect of overingestion of carbohydrate-rich diet that mainly manifests in late age, compared to a more immediate effect of more readily available protein from the equal P:C diet on mortality rate early in life.

Overall higher baseline mortality rates of females on the high-carbohydrate diet and corresponding lower median lifespan may be a result of high carbohydrate content leading to generally elevated metabolism and activity, or it might stem from the inability of females on rich carbohydrate diet to ingest sufficient amounts of protein because they would have had to massively overingest carbohydrates to meet their protein needs for elevated egg production. Female field crickets have been shown to choose a much higher protein-to-carbohydrate intake ratio of about 1:3 (Maklakov et al. 2008a), compared to a P:C ratio of 1:8 of our carbohydrate-rich diet, forcing them to either overingest carbohydrates or underingest protein to meet their intake target.

Virgin male crickets lived shorter adult lives, on average, than mated males. This might be because virgins exerted higher calling effort than mated males. While average calling effort was not affected by diet, males on a high-carbohydrate diet were more likely to senesce after reaching their peak calling effort at around 40 days of age and mated males on this diet showed much faster senescence in their age-dependent mortality rates than males on an equal P:C diet. Virgin males in our study called more than mated males, and lived shorter lives. A similar relationship between calling effort and survival has been reported in a previous study in which diet-induced variation in calling effort also dramatically altered male longevity (Hunt et al. 2004a). Whereas males in the Hunt et al. study were kept as virgins, in the present study, we were able to determine that mating, together with observed correlated lower calling effort, does not lead to equal or even more reduced lifespan compared with virgin males.

Recently, Maklakov et al. (2008a) showed that male *T. commodus* live longer and call more when given a high-carbohydrate diet (such as diet C in our study) than a diet with an even protein:carbohydrate ratio (such as diet P). Maklakov et al. (2009) likewise showed that dietary protein had little effect on age-dependent calling effort, whereas males increased their calling effort more rapidly with age when fed higher carbohydrate diets. In the present study, we targeted our diet treatment by focusing on only two diets (the male- and female-optimal diets for reproductive effort), rather than using the full geometric framework approach suggested by Simpson and Raubenheimer (2007) and used in *Drosophila* by Lee et al. (2008) and Maklakov et al. (2008a, 2009) in the same population of crickets as ours. We found a weak effect of our chosen diets on lifespan and no effect on total calling effort, unlike Maklakov et al. (2008a). But importantly, males on carbohydrate-rich diets did increase their calling effort much faster early in life, compared to males on equal P:C diet, a result that is consistent with Maklakov et al. (2009).

Furthermore, once males on the high-carbohydrate diet reach their peak calling age around day 40, their calling effort senesces, but on the high-protein diet calling effort maintains a plateau with little sign of senescence. Indeed, males only began to senesce in calling effort between 40 and 60 days of age; therefore, most of the senescence we recorded occurs after the maximum expected field lifespan of 58 days and well beyond the estimated median male age of 15 days in the wild (Zajitschek et al. 2009b). Virgin males increased their calling effort faster than mated males in early life. This fits a pattern of intensified terminal investment in reproduction, given the estimated longevity in the field, and is likely to be shaped by an invariably very strong force of natural selection to mate at least once. This does not mean that selection on mated males to mate is less strong. Mated males increase their calling effort with age as well, but this increase is not as pronounced as in virgins, which might be caused by the investment in mating that constrains investment in calling effort.

### Diet-dependent ageing

The elevated senescence in male calling effort on the high-carbohydrate diet, and of actuarial senescence exhibited by all females and by mated males on this diet (but not on the equally calorific diet P) supports the assertion that macronutrient balance rather than simple calorie content alters ageing rates (Lee et al. 2008; Skorupa et al. 2008; Ja et al. 2009; Raubenheimer et al. 2009). Although it has long been thought that ageing is accelerated on a high-calorie diet and slowed on a restricted calorie diet (Masoro 2002), it now appears that calorie content is not the crucial dietary trait that regulates responses to dietary restriction (Mair et al. 2005). Rather, the long-documented effects of dietary restriction on ageing appear to be due to the balance between macronutrients, most prominently between protein and carbohydrates (Lee et al. 2008; Ja et al. 2009; Simpson and Raubenheimer 2009; Tatar 2011). Unlike these previous studies, however, it seems that high-carbohydrate diets promote earlier and more rapid senescence than diets with an equal P:C ratio. This finding suggests not only that ageing depends on complex interactions between diet, reproductive effort, and sex, but that different species might have very different responses.

### Reproductive effort

Most studies of the effects of mating on reproduction are typically performed on females, and find that mating shortens lifespan (Partridge and Farquhar 1981; Fowler and Partridge 1989; Tatar et al. 1993; Paukku and Kotiaho 2005; Wedell 2010). This is indeed the pattern we see for females in the present study, with the survival cost of mating independent of diet. The cost of mating in female field crickets is consistent with the nature of their reproductive effort, in that mating itself causes damage as well as the transfer of costly prostaglandin-rich seminal fluid (Loher 1981; Loher et al. 1981; Tobe and Loher 1983; Murtaugh and Denlinger 1987). It is also followed by egg laying, which uses up valuable resources, including proteins (Wheeler 1996).

Unlike in females, and in most other studies of the costs of mating (Kotiaho and Simmons 2003; Paukku and Kotiaho 2005; Oliver and Cordero 2009), mated males in our study lived longer than virgins. One possible reason for this greater longevity among mated males is that those males called less than virgins. This could be an adaptive response to mating: a male who has mated knows that there are females present and willing to mate with him at his current level of investment in calling, whereas unmated males have never received this information and increase their calling effort in the absence of any feedback that they are attractive. The fact that a single mating per week can decrease male calling effort to such an extent that it prolongs lifespan (beyond any lifespan costs of the mating itself) suggests that males make complex and subtle adjustments to their resource allocation strategies in relation to their local circumstances. It also suggests that the ageing and longevity costs of calling effort might be far more substantial than the costs of mating.

A similar pattern to our results has been found in *Drosophila virilis* (Aigaki and Ohba 1984), where the average lifespan of virgin males of 11 experimental strains was 12% lower, on average, compared to mated males. Mated flies in the latter study were kept in mating groups with equal sex ratio during the whole experiment, which allows for higher mating rates compared to our experimental design, where males were allowed to mate one night a week. In redback spiders (*Latrodectus hasselti*), mated females, but not males, were found to live longer than virgin females (Stoltz et al. 2010). The authors were able to show that, in fact, resting metabolic rate was higher in virgin than in mated spiders, which could lead to the observed negative effect on survival. It remains to be seen whether mating impacts the resting metabolism of mated male field crickets, but that is certainly one possible mechanism. Generally, it seems important to note that the cost of mating is a part of the cost of reproduction, which entails other behavioral and physiological components (e.g., calling in male crickets or resting metabolism in spiders) that might have a negative effect on survival and might partly be affected by a trade-off with the cost of mating. Reproductive behavior which is not as obvious to the human experimenter (as, e.g., conspicuous acoustic sexual advertisements in male Orthoptera is) might often be neglected as a costly part of reproduction (Miyo and Charlesworth 2004).

In conclusion, we show in this study that mating and diet have profound impacts on sex-specific mortality patterns, and that these effects interact in complex ways. Our findings suggest that the search for general cross-taxon causes of the links between diet, reproduction, and ageing might yield few generalities, and that researchers should be open to the differences between species. Future research in the mechanistic factors affecting differences in baseline mortality or ageing rates will open up new insights into how and why males and females differ.

## Supporting Information

**Table S1.** Summary of Gompertz model results of males.

**Table S2.** Summary of Gompertz model results of females.

**Figure S1.** Gompertz model parameters for treatment groups and males and females.
