# Supplementary material for: Diet, sex, and death in field crickets
Source: Ecol Evol. 2012 Jul;2(7):1627–36. doi: 10.1002/ece3.288 (PMC3434943; doi:10.1002/ece3.288)
Supplement: Supplementary file 1 [file ece30002-1627-SD1.doc]

**Supporting information for Zajitschek et al.: *Diet, sex and death in field crickets***

***Content***

S1 (Table). Summary of Gompertz model results of males.

S2 (Table). Summary of Gompertz model results of females.

S3 (Figure). Gompertz model parameters for treatment groups and males and females.

S1. Summary of Gompertz model results of males. Models are ordered with the best-fit model at the top. For each Gompertz α and *β*, variables (mated, diet) are given, for which α and *β* were allowed to take on separate values. The Gompertz model ‘No treatment effects’ is fitted independently of variables mated and diet. The ‘No senescence’ model fits a constant hazard rate. Interaction terms are depicted with a cross (mated × diet), two main effects with a plus (mated + diet). Models with ΔAIC ≤ 2 are shown above the dotted line.

| # | Model | *α* | *β* | df | ΔAIC | AIC weight |
| --- | --- | --- | --- | --- | --- | --- |
| 1 | *α* Diet_ *β* (Diet+Mated) | Diet | Diet + Mated | 5 | 0 | 0.239 |
| 2 | *α* Diet_ *β* (Diet×Mated) | Diet | Diet × Mated | 6 | 0.1 | 0.231 |
| 3 | *α* (Diet+Mated)_ *β* Mated | Diet + Mated | Mated | 5 | 0.7 | 0.171 |
| 4 | *α* *β* (Diet+Mated) | Diet + Mated | Diet + Mated | 6 | 1.8 | 0.100 |
| 5 | *α* *β* (Diet×Mated) | Diet × Mated | Diet × Mated | 8 | 2.4 | 0.073 |
| 6 | *α* (Diet×Mated)_ *β* Mated | Diet × Mated | Mated | 6 | 2.4 | 0.072 |
| 7 | *α* (Diet+Mated) | Diet + Mated |  | 4 | 3.9 | 0.035 |
| 8 | *α* (Diet×Mated) | Diet × Mated |  | 5 | 4.2 | 0.029 |
| 9 | *α* (Diet+Mated)_ *β* Diet | Diet + Mated | Diet | 5 | 4.6 | 0.024 |
| 10 | *α* (Diet×Mated)_ *β* Diet | Diet × Mated | Diet | 6 | 4.9 | 0.020 |
| 11 | *α* Diet | Diet |  | 3 | 9.9 | 0.002 |
| 12 | *α* *β* Diet | Diet | Diet | 4 | 10.1 | 0.002 |
| 13 | *β* (Diet×Mated) |  | Diet × Mated | 5 | 10.3 | 0.001 |
| 14 | *β* (Diet+Mated) |  | Diet + Mated | 4 | 11.8 | < 0.001 |
| 15 | *α* Mated_ *β* (Diet×Mated) | Mated | Diet × Mated | 6 | 12.2 | < 0.001 |
| 16 | *α* Mated_ *β* (Diet+Mated) | Mated | Diet + Mated | 5 | 13.8 | < 0.001 |
| 17 | *β* Diet |  | Diet | 3 | 23.3 | < 0.001 |
| 18 | *β* Mated |  | Mated | 3 | 31.4 | < 0.001 |
| 19 | *α* Mated | Mated |  | 3 | 33.3 | < 0.001 |
| 20 | *α* *β* Mated | Mated | Mated | 4 | 33.4 | < 0.001 |
| 21 | No treatment effects |  |  | 2 | 38.8 | < 0.001 |
| 22 | No senescence |  |  | 1 | 203.3 | < 0.001 |

S2. Summary of Gompertz model results of females. Models are ordered with the best-fit model at the top. For each Gompertz α and *β*, variables (mated, diet) are given, for which α and *β* were allowed to take on separate values. The Gompertz model ‘No treatment effects’ is fitted independently of variables mated and diet. The ‘No senescence’ model fits a constant hazard rate. Models with ΔAIC ≤ 2 are shown above the dotted line.

| # | Model | *α* | *β* | df | ΔAIC | AIC weight |
| --- | --- | --- | --- | --- | --- | --- |
| 1 | *α* Diet | Diet |  | 3 | 0 | 0.181 |
| 2 | *β* Diet |  | Diet | 3 | 1 | 0.110 |
| 3 | *α β*Diet | Diet | Diet | 4 | 1.7 | 0.077 |
| 4 | *α* (Diet+Mated)_ *β* Mated | Diet + Mated | Mated | 5 | 1.7 | 0.077 |
| 5 | *β* (Diet×Mated) |  | Diet × Mated | 5 | 1.8 | 0.073 |
| 6 | *α* (Diet+Mated) | Diet + Mated |  | 4 | 2 | 0.067 |
| 7 | *α* Mated_ *β* (Diet×Mated) | Mated | Diet × Mated | 6 | 2 | 0.067 |
| 8 | *β* (Diet+Mated) |  | Diet + Mated | 4 | 2.4 | 0.060 |
| 9 | *α* Diet _ *β* (Diet×Mated) | Diet | Diet × Mated | 6 | 2.9 | 0.042 |
| 10 | *α* (Diet×Mated) | Diet × Mated |  | 5 | 2.9 | 0.042 |
| 11 | *α* (Diet×Mated)_ *β* Mated | Diet × Mated | Mated | 6 | 3.1 | 0.039 |
| 12 | *α* Mated_ *β* (Diet+Mated) | Mated | Diet + Mated | 5 | 3.2 | 0.037 |
| 13 | *α* Diet _ *β* (Diet+Mated) | Diet | Diet + Mated | 5 | 3.2 | 0.036 |
| 14 | *α* *β* (Diet+Mated) | Diet + Mated | Diet + Mated | 6 | 3.4 | 0.033 |
| 15 | *α* (Diet+Mated)_ *β* Diet | Diet + Mated | Diet | 5 | 3.7 | 0.028 |
| 16 | *α* (Diet×Mated)_ *β* Diet | Diet × Mated | Diet | 6 | 4.6 | 0.018 |
| 17 | *α* *β* (Diet×Mated) | Diet × Mated | Diet × Mated | 8 | 5 | 0.015 |
| 18 | No treatment effects |  |  | 2 | 8.7 | 0.002 |
| 19 | *β* Mated |  | Mated | 3 | 10.6 | < 0.001 |
| 20 | *α* Mated | Mated |  | 3 | 10.7 | < 0.001 |
| 21 | *α* *β* Mated | Mated | Mated | 4 | 12 | < 0.001 |
| 22 | No senescence |  |  | 1 | 143.3 | < 0.001 |

S3. Gompertz model parameters for treatment groups and males (a) and females (b). Error bars indicate 95 % confidence intervals. Parameters were estimated in separate models on specified subgroups of experimental animals. We also present parameter estimates from models of data on all males and females (All).
